# Transfer RNA (tRNA) Genes, Codon Usage and Translational Efficiency in *Leishmania infantum*

**DOI:** 10.3390/genes17060620

**Published:** 2026-05-29

**Authors:** Ariel Nájera-Peso, Andrés Carrazco-Montalvo, Javier Adán-Jiménez, Jose M. Requena

**Affiliations:** 1 Centro de Biología Molecular Severo Ochoa (CSIC-UAM), Departamento de Biología Molecular, Instituto Universitario de Biología Molecular (IUBM), Universidad Autónoma de Madrid, 28049 Madrid, Spain; arielnnajera@gmail.com (A.N.-P.); andres.carrazco@estudiante.uam.es (A.C.-M.); javier.adan@cbm.csic.es (J.A.-J.); 2 Centro de Investigación Biomédica en Red de Enfermedades Infecciosas, Instituto de Salud Carlos III, 28029 Madrid, Spain

**Keywords:** *Leishmania*, transfer RNAs, tRNAs, proteome, codon usage, protein expression

## Abstract

**Background/Objectives**: Protozoan parasites of the genus *Leishmania* are causative agents of a group of devastating human diseases, known as leishmaniasis. These microorganisms possess very unusual mechanisms of gene expression that are poorly understood. This study was aimed at analyzing the tRNA repertoire encoded in the *Leishmania infantum* genome, a species responsible for the most severe form of disease, visceral leishmaniasis. tRNAs are adaptor molecules aimed at decoding mRNAs into proteins. **Results**: A total of 92 tRNA genes, dispersed on 38 loci, were identified, often located in regions where unidirectional gene arrays converge. Putative intronic sequences were inferred for three tRNA genes, and, remarkably, nine tRNAs were found to overlap with the protein-coding sequences of annotated genes. According to structural predictions, the *L. infantum* tRNA repertoire covers 49 out of the 61 possible anticodons, but because of the well-documented wobble phenomenon, these are enough to decode all codons in the 8532 protein-coding genes currently annotated in its genome. As illustrated in this study, codon usage is a well-conserved trait among different *Leishmania* species but it differs substantially regarding its human host. Finally, we analyzed tRNA adaptation index (tAI) parameters, codon usage metrics, and relative protein expression levels. **Conclusions**: Apart from providing the tRNA gene repertoire and its genome distribution, we have shown the existence of a statistically significant, positive correlation between the tAI scores and protein expression levels in *L. infantum* promastigotes.

## 1. Introduction

The molecular biology central dogma explains how genetic information (stored as DNA/RNA) is expressed as amino acid sequences (proteins) by a process known as translation that takes place in a supramolecular machine called a ribosome [1]. The ribosome reads the genetic information transcribed into messenger RNAs (mRNAs) one codon (three nucleotides) at a time to insert the appropriate amino acid into a polypeptide chain using adaptor molecules (aminoacyl-tRNAs) that carry a particular amino acid and recognize a specific codon by base pairing with their anticodon. There are 64 possible codons, but three of them usually act as stop codons because there are no tRNAs that can interact with them; stop codons mark where translation should terminate. On the other hand, there are 20 different amino acids in natural proteins and, therefore, tRNAs with different anticodons can be loaded with the same amino acid; this class of tRNAs are known as isoacceptors [2]. On the contrary, in a given organism, not all of the 61 encoding codons have a dedicated tRNA, but a single tRNA anticodon may recognize several codons due to wobble base pairing; thus, tRNAs with the same anticodon (able to decode the same set of codons) are named isodecoders. Because of wobble, the minimal number of different tRNAs needed to decode all 61 codons is 32 [2]. However, usually, the number of isodecoders is higher than 32 and varies in the different organisms; moreover, each isodecoder can be present in multiple gene copies. Thus, in model eukaryotes, the number of tRNA genes varies between 170 and 570, and the number of different tRNA isodecoders ranges from 41 to 55; usually the number of tRNA genes increases in parallel to the cellular complexity of organisms [3]. However, it is worthy to note that tRNAs with particular translational-active anticodons are absent in all kingdoms of life: bacteria, archaea, and eukaryotes [4].

*Leishmania* parasites belong to the group of kinetoplastids that are among the earliest-branching eukaryotes [5]. A peculiarity of these parasites is the absence of transcriptional control on a per-gene basis for protein-coding genes, as post-transcriptional mechanisms operate on the mRNA translational efficiency are key for controlling gene expression [6,7]. At least two molecular factors are particularly relevant to determining the translational efficiency of a given mRNA coding sequence, the codon usage and the tRNA repertoire. After having available the first genome sequence for a *Leishmania* species, Padilla-Mejía and co-workers identified 83 tRNA-coding genes in the *Leishmania major* genome, a relatively lower number of genes compared to those in model organisms [8]. Although the genomes for other *Leishmania* species have been sequenced, an inventory of tRNA genes existing in species other than *L. major* has not been reported to date.

Codon usage refers to the preferential or non-random use of synonymous codons. Because of genetic code degeneracy, the same amino acid is incorporated after reading different codons (namely synonymous codons). Apart from tryptophan and methionine, each one encoded by a single codon, the remaining 18 amino acids are encoded by several synonymous codons. The usage with different frequencies of synonymous codons is termed codon usage bias (CUP), and different species have consistent and characteristic CUPs. Moreover, codon usage bias varies not only between organisms but also between groups of genes within a particular organism [9]. Codon usage is a relevant regulator of gene expression as the translational rate of an mRNA would depend on, among other factors, the abundance of the tRNAs needed to decode its coding sequence. In general, highly expressed genes contain preferred codons whose frequencies correlate with the abundances of the tRNA isoacceptors in a particular organism [10]. The relevance of this evolutionarily balanced adaptation in the codon preference of particular genes has been extensively documented in humans, where single mutations in coding regions that do not change the coded amino acids (i.e., synonymous mutations) have been linked to disease and cancer development [11].

The codon usage of three *Leishmania* species (*L. major*, *L. infantum* and *Leishmania braziliensis*) was reported more than ten years ago [12], when the genome assemblies, particularly those for *L. infantum* and *L. braziliensis*, were incomplete. In fact, at that time, only 66 tRNAs could be identified in the *L. infantum* genome. In 2017, an improved assembly for the *L. infantum* genome was obtained by González-de la Fuente and co-workers [13]. Based on this improved genome, and using two complementary bioinformatics tools, we identified a total of 92 tRNAs genes. According to their anticodon sequences, they cover a total of 49 different triplets (codons). Considering this repertoire of tRNAs and the wobble rules, the translational adaptation index (tAI) was calculated for each of the currently annotated proteins in this *Leishmania* species. Finally, by analyzing the relative abundance of the proteins identified in the *L. infantum* promastigotes, a statistically significant correlation between tAI values and protein abundance was observed.

## 2. Materials and Methods

### 2.1. Leishmania Genomes and Protein-Coding Sequences

The genome sequences and protein-coding sequences used in this study are publicly available at the Leish-ESP repository (https://jmrequenajmr.wixsite.com/leish-esp, accessed on 11 April 2026).

### 2.2. tRNA Gene Annotation

Two bioinformatics programs were used: tRNAscan-SE 2.0.12 [14] and ARAGORN 1.2.41 [15]. To ensure maximum sensitivity, several search modes (eukaryotic, mitochondrial, organellar, and COVE) were selected when the tRNAscan-SE program was run. The ARAGORN program was downloaded through the following link: https://www.trna.se/ (accessed on 11 April 2026). The results obtained through the different searches were merged to generate a non-redundant list of tRNA genes. When predictions of anticodons were not coincidental in both programs, we underwent visualization of the predicted structures looking for the more accurate anticodon based on the secondary structure rules underpinned by Rich and RajBhandary [16]. Finally, genomic coordinates for tRNAs were refined according to their predicted secondary structures.

### 2.3. Analysis of Codon Usage in Protein-Coding Sequences

A bioinformatic tool was developed to determine the absolute and relative numbers of codons found in every coding sequence (CDS). This tool was designed to be used in a friendly manner at the following website: https://leishmania.cbm.uam.es/tools/codon-usage (accessed on 11 April 2026). Codon usage was determined for the following species (datasets): *L. infantum* (https://data.mendeley.com/datasets/f9sr5bgv24/1, accessed on 11 April 2026), *L. major* (https://data.mendeley.com/datasets/b299x68yj7/1, accessed on 11 April 2026), *L. braziliensis* (https://zenodo.org/records/19205547, accessed on 11 April 2026), and *Homo sapiens* (https://zenodo.org/records/18787765, accessed on 11 April 2026).

For principal component analysis (PCA), the codon usage frequencies for the 64 codons were arranged in a matrix with codons as rows and species as columns. Principal component analysis was conducted in R (v4.5.0) using the prcomp() function after transposing the matrix so that species were treated as observations and codon frequencies as variables. For the heatmap, codon frequencies were normalized by row using Z-score, and hierarchical clustering was applied to both codons and species using the pheatmap package. Data visualizations, including PCA biplots and scatter plots with LOESS, smoothing were generated using the ggplot2 (v3.4.0) package.

### 2.4. Calculation of the tRNA Adaptation Index (tAI)

The tAI for every *L. infantum* protein-coding gene was calculated according to dos Reis and coworkers [17]. For each codon, an adaptiveness numerical value, Wik, that takes into account the number of tRNA isoacceptors that recognize a given codon and the efficiency of the codon–anticodon interaction based on the Crick’s wobble rules for codon–anticodon pairing was estimated [17]. Wi values were normalized according the maximum Wi value (taken arbitrarily as 1) among those coding for the same amino acid. Finally, the tAI of a coding sequence was calculated as the geometric mean of the relative adaptiveness values of its codons. For calculations, we used the formula:

$${tAI}_{g}=exp\left(\frac{1}{L_{g}}\sum_{k=1}^{L_{g}}\ln\left(w_{i_{k}}\right)\right)$$

where the tAI_g_ (i.e., tAI for a given gene (g)) is defined as the geometric mean of the relative adaptation weights (wᵢₖ) of the number of codons (Lg) that compose the gene. The formula is expressed in a logarithmic form to ensure numerical stability during computation. In sum, the tAI is a measure of the adaptation of a given gene to the available tRNA pool.

### 2.5. Parasite Culture, Protein Samples and Proteomic Data

Promastigotes of *L. infantum* JPCM5 strain were grown at 26 °C in Roswell Park Memorial Institute (RPMI) medium supplemented with 15% of heat-inactivated fetal bovine serum (FBS), hemin (10 μg/mL) and an antibiotic mix (streptomycin 10 μg/mL and penicillin 10^5^ U/mL). Cultures (50 mL) were started at 5 × 10^5^ cells/mL and the parasites were harvested in the middle logarithmic growth phase (10^7^ promastigotes per mL). After washing twice with phosphate-buffered saline (PBS), the pellets (5 × 10^8^ cells) were processed using the Subcellular Protein Fractionation Kit for Cultured Cells (Cat. Number 78840; Thermofisher Scientific, Waltham, MA, USA). As a result, four fractions were obtained and processed for proteomic analysis.

Each fraction was submitted to in-gel digestion using sequencing grade trypsin (Promega, Madison, WI, USA) following the procedure described elsewhere [18]. Peptide samples were analyzed by reverse phase–liquid chromatography (RP-LC)-MS/MS analysis (Dynamic Exclusion Mode) in an Easy-nLC 1200 system coupled to an ion trap LTQ-Orbitrap Velos Pro hybrid mass spectrometer (Thermo Scientific, Waltham, MA, USA). The proteome raw data are publicly available at Zenodo repository (https://zenodo.org/records/18639527, accessed on 11 April 2026). Peptide identification from raw data was carried out using the PEAKS Studio XPro search engine (Bioinformatics Solutions Inc.,Waterloo, ON, Canada) [19]. Searches were performed against the most recent *L. infantum* proteome dataset available at the Mendeley Data server (https://data.mendeley.com/datasets/dtmstvb2j5/2, accessed on 11 April 2026).

### 2.6. Calculation of Relative Protein Abundance

From the peptide mass spectra associated with the *L. infantum* (JPCM5 strain) proteome, we determined the relative protein abundance using the “Top3” method, which sums the intensities of the three most abundant unique peptides identified for each protein [20].

## 3. Results and Discussion

### 3.1. Annotation of tRNA Genes in the L. infantum Genome

A search for tRNA genes was conducted on the more recent genome sequence of the reference strain (JPCM5) for *L. infantum* [13] by using two bioinformatics programs: tRNAscan-SE 2.0.12 [14] and ARAGORN 1.2.41 [15]. The ARAGORN algorithm combines a search of tRNA consensus sequences together with their ability to conform a secondary structure adapting the typical cloverleaf form. The detection sensitivity of this program was estimated to be 99% in sequences from all three life kingdoms [15]. The tRNAscan-SE algorithm is trained with large sets of clade-specific tRNAs and specialized isotypes, being able to identify typical tRNAs but also to discriminate atypical ones like initiator methionine tRNA (iMet tRNA), elongator methionine tRNA (Met tRNA) and selenocysteine tRNA [14]. Although ARAGORN and tRNAscan-SE yielded coincidental results for most identified tRNAs, a few discrepancies were also observed (see below). As expected, ARAGORN and tRNAscan-SE have been demonstrated to be confident tools for tRNA identification in *Leishmania*, but it is recommended to use both as they complement each other in the characterization of atypical tRNA structures. As a result of this analysis, 92 tRNAs were predicted in the *L. infantum* (JPCM5) genome (see Table 1 and Supplementary File S1). In a previous study, 83 tRNA genes were identified in the *L. major* genome [8], which is quite similar to the number reported here.

Two of the tRNAs were predicted to be iMet tRNAs (LINF_090013000 and LINF_360074700) and another two coding for internal Met tRNAs (LINF_110015000 and LINF_340017200). For structural details allowing to differentiate both types of Met tRNAs, see the article by Padilla-Mejia and co-workers [8]. Also, a specialized tRNA for introducing selenocysteine (SeC tRNA) was identified (LINF_060007300). The existence of selenoproteins, and the factors required for their translation, has been documented in *L. major* [21].

**Figure 1:**
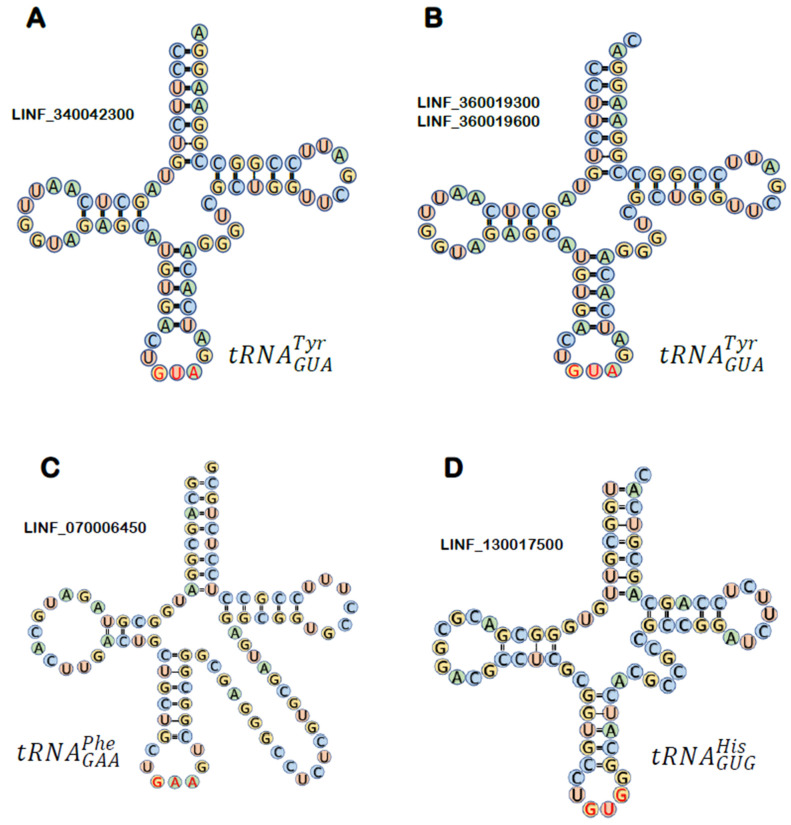
Cloverleaf representation of several *L. infantum* tRNAs. (**A**) LINF_340042300; (**B**) LINF_360019300 and LINF_360019600 (both tRNAs have identical nucleotide sequence); (**C**) LINF_070006450; (**D**) LINF_130017500. Colored in red are the nucleotides conforming the anticodon.

A discrepancy regarding anticodon assignment between the two prediction programs occurred for three tRNAs: LINF_340042300, LINF_360019300, and LINF_360019600 (the latter two have identical nucleotide sequences). Whereas tRNAscan-SE predicted anticodons for leucine (Leu), the structure modeled by ARAGORN supports that these tRNAs would be decoding tyrosine (Tyr) (Figure 1A,B). The reason for this discrepancy in the predicted anticodons between both programs is that ARAGORN detected putative introns in the sequences of these three genes, whereas tRNAscan-SE did not. In Figure 1 (panels A and B), the secondary structures corresponding to the spliced tRNAs are shown; in Supplementary Figure S1, the anomalous tRNA structures that are predicted when the intronic sequences are included are shown. The presence of introns in tRNA genes is not a surprising finding as tRNA genes containing introns have been described in all three kingdoms of life (see [2] and references therein). In fact, tRNA^Tyr^ was also postulated to be the only intron-containing tRNA identified in the *Trypanosoma cruzi* genome [22]. Furthermore, an extremely persuasive argument in favor of the plausible structure depicted for these tRNAs is that no other *L. infantum* tRNA was found to be Tyr-specific (Table 1).

According to the structural predictions, there are two tRNAs whose structures have some deviations from the typical tRNA structure: LINF_070006450 (Figure 1C) and LINF_130017500 (Figure 1D). These tRNAs were only detected by ARAGORN. LINF_070006450 was predicted to decode phenylalanine, but it possesses a V-loop that is unusually large and the A-stem has eight nucleotides instead of seven (more common in typical tRNAs). The anomaly found in the tRNA structure predicted for LINF_130017500 is that the C-loop contains only seven nucleotides when more often this loop consists of eight bases. As a consequence, the search algorithm did not make an anticodon assignment; nevertheless, according to the consensus rule for the anticodon placement (YUNNNR [Y: C or U (pyrimidines); R: A or G (purines); NNN, anticodon]), this would be GUG, i.e., a tRNA specific for decoding histidine.

Table 1 shows the tRNAs ordered according to the decoded amino acid and its anticodon. Forty-nine out of the sixty-one theoretically possible decoding tRNAs were identified in the *L. infantum* genome. Nevertheless, as also shown in Table 1, all the 61 codons were found to exist among the annotated protein-coding sequences in this parasite. We will come back to this question in Section 3.4.

### 3.2. Genomic Organization of the tRNA Genes

**Figure 2:**
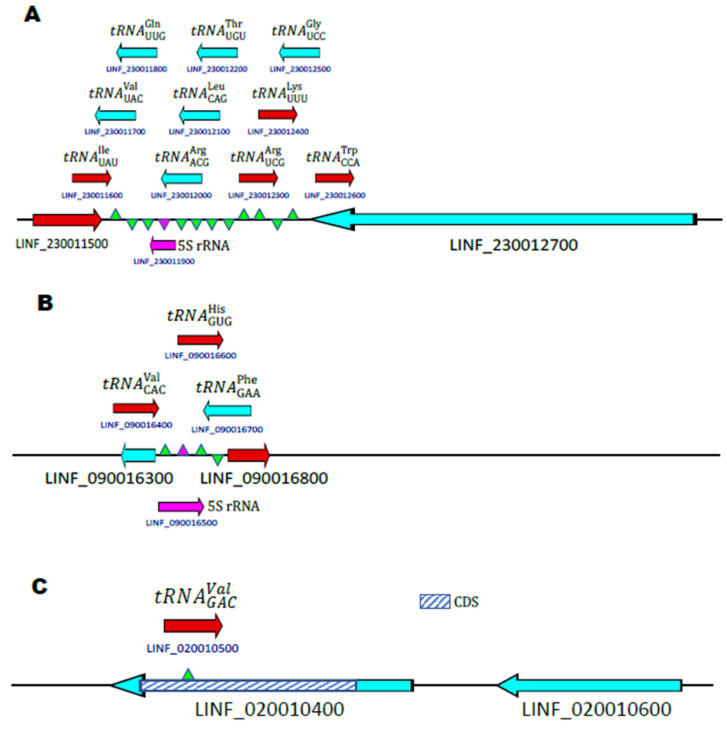
Organization of tRNA genes at particular loci in the *L. infantum* genome. (**A**) The locus with the larger number of tRNAs (ten) is located at chromosome 23. These genes are in a region in which two transcriptional units converge. (**B**) A locus in chromosome 9 containing three tRNAs within a region in which two transcriptional units diverge. (**C**) Example of a tRNA (LINF_020010500) overlapping with the protein-coding sequence of gene LINF_020010400. Protein-coding genes and intergenic regions are drawn to scale, but not the genes coding for tRNAs and 5S rRNA (pink arrows). The positions of the tRNAs on the chromosome are shown by green triangles, and above them the corresponding tRNAs are depicted. Red or blue arrows indicate that genes are located at the plus or minus strand, respectively. The ID codes for every gene are included, and for tRNAs the anticodon and decoded amino acid are shown.

The 92 tRNA genes identified in the *L. infantum* species are distributed on 23 out of the 36 chromosomes comprising the genome (detailed information is provided in the Supplementary File S1). The tRNA genes are found either alone or grouped, amounting to a total of 38 different loci. The locus with a large number of tRNA genes is located at chromosome 23 and it comprises 10 tRNA genes and a 5S rRNA gene (Figure 2A). Remarkably, this gene locus is located at the chromosomal point in which two transcriptional units converge, suggesting that the tRNA loci might serve as stopping points for the RNA polymerase II transcriptional machinery. There are other tRNA loci located in equivalent positions (i.e., confluence of transcriptional units) at chromosomes 3, 5, 9, 15, 16, 21 (two loci), and 24. Although less frequently, some tRNA loci are found between divergent transcriptional units in chromosomes 9 (Figure 2B) and 10.

Another remarkable finding is the location of some tRNA genes within coding sequences (CDS) of protein-coding genes. An example is tRNA LINF_020010500, whose sequence overlaps with the CDS of gene LINF_020010400 but in an antisense orientation regarding the CDS (Figure 2C). This gene encodes a large protein (2091 amino acids in length) without any distinctive structural domain, but is highly conserved among different *Leishmania* species (see UniProt entry A0A6L0WHP6 for further details). Other tRNA genes overlapping with protein-coding sequences are as follows: LINF_070006450 (located in sense orientation within the CDS of gene LINF_070006400, which encodes for a TMEM115/Pdh1/Rbl19-like protein); LINF_130017500 (located in antisense within the CDS of gene LINF_130017400, which encodes a thiamin pyrophosphokinase [23]); LINF_180013900 (in the sense strand within the LINF_180013800 CDS, which encodes a protein of unknown function (869 amino acids in length) that is conserved among different *Leishmania* species; LINF_210019700 (in sense orientation within the LINF_210019800 CDS, and the encoded protein contains a conserved leucine-rich repeat domain); LINF_280009850 (in antisense orientation within the LINF_280009800 CDS, which encodes a NuSAP1-like protein); and LINF_290019900 (in antisense orientation within the LINF_290019800 CDS, which codes for an RNA binding protein). This finding is not an absolute novelty, as the presence of a tRNA within a CDS was previously described in *Trypanosoma brucei*, a *Leishmania*-related trypanosomatid, by Padilla-Mejía and co-workers [8]. An open question is whether these overlapping tRNAs are functional, and the response will be obtained after the specific isolation of small RNAs and their massive sequencing.

### 3.3. Analysis of Codon Usage in L. infantum Protein-Coding Sequences

The frequency of codons was calculated using an in-house Python script (see Section 2). Currently, the number of protein-coding genes annotated in the *L. infantum* (JPCM5 strain) is 8532 (https://data.mendeley.com/datasets/f9sr5bgv24/1, accessed on 11 April 2026). Table 1 shows the relative frequencies (per thousand) for each of the 61 sense codons. In the Supplementary File S1, individual codon frequencies for every protein-coding sequence are provided. Regarding the stop codons, the less-used non-sense (or termination) codon is TAA (20.5%) followed by TAG (36.6%) and TGA (42.9%). A factor that may contribute to those differences is the high G+C content (59.74%) of the *L. infantum* genome. In agreement with this high G+C content, among the synonymous codons, those having a GC-richness are clearly more abundant (Table 1). For instance, the Arg-coding codon CGC is around 12-fold more frequent than codon AGA.

**Figure 3:**
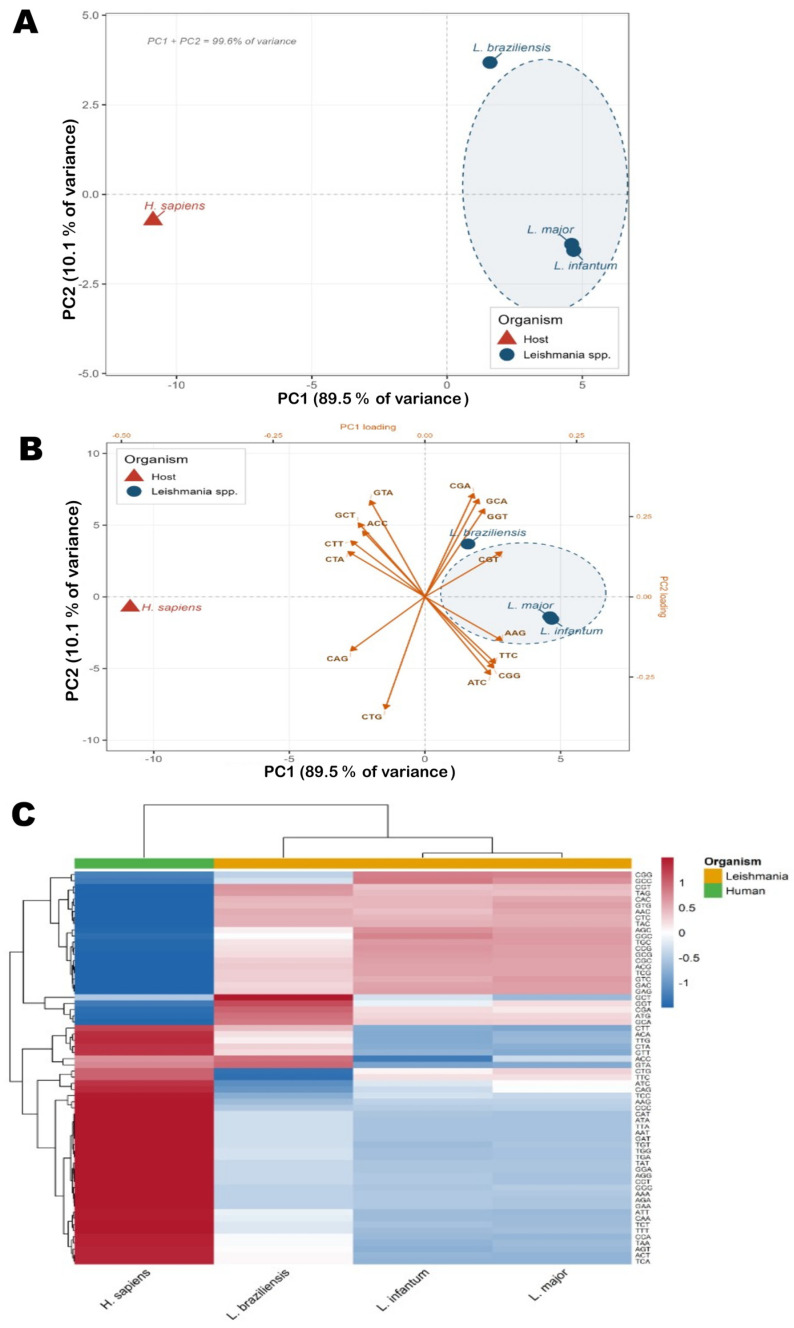
Comparison of codon usage between three *Leishmania* species and its human host. (**A**) Principal component analysis (PCA) based on the Relative Synonymous Codon Usage (RSCU) values of the 61 sense codons. The dashed blue ellipse represents the 95% confidence interval for the *Leishmania* group; (**B**) PCA biplot illustrating codon loadings and species separation. The biplot overlays species scores with the loading vectors (orange arrows) of the 15 codons contributing most significantly to the variance. (**C**) Hierarchical clustering heatmap based on row-wise Z-score normalized codon frequencies. Red indicates relatively higher codon usage, blue indicates relatively lower codon usage, and white indicates values close to the mean for each codon.

To determine possible codon usage variations along the genus *Leishmania*, we also analyzed the codon frequencies in the species *L. major*, belonging to the *Leishmania* subgenus as *L. infantum*, and *L. braziliensis*, as representative of the *Viannia* subgenus. Despite the 20–100 million years of divergence existing between these *Leishmania* species [24], a strong conservation in codon usage was observed across the genus (Figure 3A–C). For comparative purposes, the codon usage in *H. sapiens* was also determined, showing clear differences with that found in the *Leishmania* coding sequences. The PCA (Figure 3A) showed a clear separation between *H. sapiens* and the *Leishmania* species, mainly along PC1, which explained most of the variation in codon usage. Together, PC1 and PC2 explained 99.6% of the total variance, indicating that the two-dimensional PCA captured almost all of the differences among the analyzed species. *Leishmania* species were located in a distinct region of the PCA space compared with human species. Within *Leishmania*, *L. infantum* and *L. major* showed the closest similarity, whereas *L. braziliensis* appeared more distinct along the PC2 axis. Figure 3B shows the fifteen codons contributing most significantly to the variance.

The hierarchical clustering heatmap analysis (Figure 3C) evidenced similar conclusions to the PCAs. *H. sapiens* displayed a codon usage profile that differed from the *Leishmania* species, while again *L. infantum* and *L. major* clustered more closely together. The heatmap also allowed a clearer comparison of individual codons, showing codons that were relatively more or less used in each species. Overall, these analyses show a clear host–parasite separation in codon usage profiles, while also revealing differences among the two *Leishmania* subgenus *Leishmania* and *Viannia*. Thus, *H. sapiens* shows preference for A/T-rich codons whereas *Leishmania* species prefer G/C-rich codons. In a previous study, a comparison of the codon usage between *Leishmania* genomes and other related trypanosomatids showed that codons rich in G or C were more preferred in *Crithidia* and *Leishmania* species than in species of the genus *Trypanosoma* [25].

### 3.4. Determination of the Translational Efficiency of the L. infantum Protein Coding Genes

As discussed in previous sections, the relative usage of synonymous codons is conditioned by the overall G+C content of a given genome. In this regard, it would be expected that the tRNA pool found in a given organism mirrors codon frequencies of its protein-coding genes. However, this is not an absolute rule in *L. infantum* (Table 1), and a remarkable example is found in the Ile-coding codons: the more frequent codon, ATC (frequency, 18.91‰), does not have a specific decoding tRNA, whereas the other two codons, which possess dedicated tRNAs, ATT (with three decoding tRNAs) and ATA (with one decoding tRNA) are present at lower frequencies, 8.37 and 2.72, respectively. As mentioned above, the lack of a given anticodon-containing tRNA it not a problem because other tRNAs are able to recognize several codons due to the flexible base-pairing between the first nucleotide of the anticodon and the third position of the codon, which is known as tRNA wobble ([17] and references therein); in other words, a tRNA may bind multiple synonymous codons, and a particular codon can be decoded by several tRNA isoacceptors.

The values compiled in Table 1 represent a mean value from all *L. infantum* protein-coding genes and we wondered whether particular sets of genes have codon usages more adapted to the tRNA pool. For this purpose, the tRNA adaptation index (tAI), which is a measure of the codon adaptation to the relative tRNA abundances [17], was calculated for each *L. infantum* protein-coding gene (values are provided in the Supplementary File S1). Among the *L. infantum* protein-coding genes, the tAI values varied from 0.8935 (the highest value, found in the gene LINF_350023700 that codes for ribosomal protein uL18) to 0.4466 (the lowest, for gene LINF_220005100, which is annotated as a putative pseudogene encoding a hypothetical protein).

To determine whether those protein-coding genes with high tAI values are indeed coding for proteins attaining high steady-state levels in the parasite, we analyzed the relative protein abundance in *L. infantum* promastigotes. For this purpose, protein extracts were used to identify and quantify peptides by liquid chromatography coupled with mass spectrometry (LC-MS), as detailed in the Materials and Methods section. To determine the relative abundance of the identified proteins, we followed an approach based on the peptide peak intensities [20]. In particular, we used the top-three method consisting of summing the intensity of the top-three most abundant peptides for a given protein. Although the methodology is very simplistic, it is based on the experimental demonstration that there exists a robust relationship between MS signals and protein concentration [26]. Thus, for every protein identified in the *L. infantum* protein extracts, the intensities of the top three most abundant peptides were summed; consequently, proteins identified by less than three distinct peptides were excluded from the analysis. Finally, 2088 of the identified proteins met the criteria (see Supplementary File S1) and were used for further analysis. The reliability of this protein quantification method is supported by the finding of, among the more abundant proteins, proteins like tubulins, heat shock proteins HSP70 and HSP90, GP63/leishmanolysin protease, and ribosome components.

**Figure 4:**
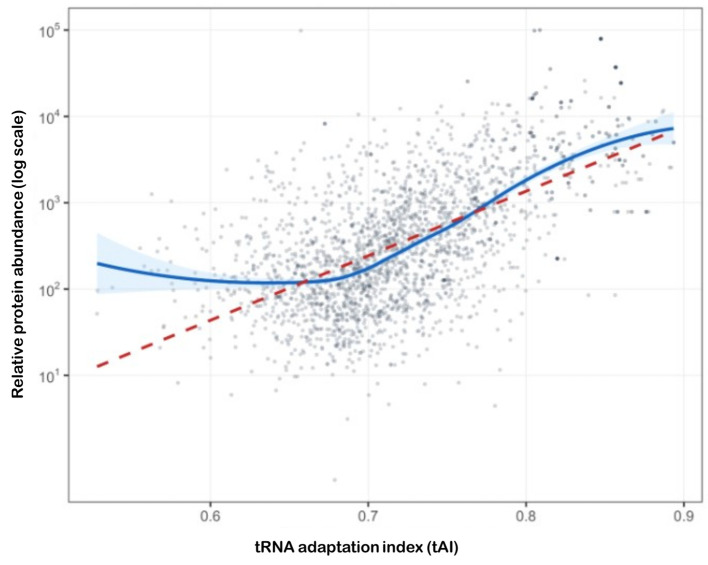
Relationship between tRNA Adaptation Index (tAI) and protein abundance in *L. infantum*. Scatter plot correlating the translational efficiency (tAI) of protein-coding genes with experimental protein levels (log_10_ scale) in *L. infantum* promastigotes (n = 2088). The solid blue line represents a locally estimated scatterplot smoothing (LOESS) curve (span = 0.75), with the shaded area indicating the 95% confidence interval of the fit. The dashed red line represents the global regression trend. A statistically significant positive association was observed (Spearman correlation coefficient, ρ = 0.582; *p* < 2.2 × 10^−16^), demonstrating that codon optimization relative to the tRNA pool would be a major determinant of protein expression levels in the parasite.

Figure 4 shows a positive correlation between tAI values and the relative abundance of the 2088 proteins quantified from the proteomic samples, as determined by Spearman’s test. This analysis showed a correlation coefficient (rho) of 0.582 (red line) with a *p* value lower than 2.2 × 10^−16^. In conclusion, this analysis evidenced a significant codon adaptation in the gene sequences coding for those proteins with a higher abundance in *L. infantum* promastigotes. In this study, we used the top-three method for label-free protein quantification, an approach that might underestimate differences at the extremes of the distribution (i.e., proteins showing very low or very large abundances). Therefore, additional proteomics data (also derived from amastigotes) should be analyzed with alternative protein quantification methods to determine how robust the relationship is.

## 4. Conclusions

A total of 92 tRNA-encoding genes have been identified in *L. infantum*. According to their anticodon, they are grouped into 49 different isodecoders (tRNAs with the same anticodon). As expected, there are 21 types of isoacceptors (tRNAs that load the same amino acid), one for each of the 20 standard amino acids and one for selenocysteine. There are dedicated tRNAs for incorporating either the initial methionine or the internal ones; two of each type were identified. Genes coding for tRNAs are dispersed on the genome, but are frequently found in genomic regions where unidirectional gene arrays converge.

It is well-accepted that particular patterns of codon usage can control ribosome speed and, consequently, the efficiency of protein synthesis [27]. In this study, we applied the statistics method developed by dos Reis and collaborators [17], supported by experimental data, that allows to calculate adaptiveness value Wi for each codon taking into account the tRNA gene pool. Thus, for a given gene, it is possible to calculate the tRNA adaptation index (tAI) as the geometric mean of the relative adaptiveness values (Wi) of its codons. When tAI values of genes and relative expression levels of the encoded proteins were compared, a strong positive relationship between the use of codons with high adaptiveness and the protein abundance in *L. infantum* promastigotes was determined.

Apart from the classical known function of tRNAs as decoders of the genetic code, in recent studies an increasing number of new functions are being attributed to them, such as regulation of transcription and translation and protein labeling for degradation [2]. Additionally, regulatory functions played by small RNA fragments derived from tRNA molecules have been involved in a variety of functions affecting chromatin structure, DNA replication and cell fate [4]. Another unexplored aspect of *Leishmania* is the chemical modifications that tRNA molecules undergo before being functional. In other organisms, more than fifty chemical modifications have been discovered in tRNAs, including methylation, pseudouridylation, acetylation, deamination, thiolation, and queuosinylation, among others (reviewed in [28]). Chemical modifications contribute to the folding and stability of tRNAs and regulate decoding capacity (especially the wobble pairing) during codon–anticodon interaction. While these research areas are waiting to be explored in *Leishmania*, this work is contributing to the identification of the tRNA gene compendium existing in this parasite, paving the road for future studies on *Leishmania* tRNA metabolism.

## Figures and Tables

**Table 1:** tRNA genes, codon W_ik_ values, and codon usage in *L. infantum*.

Amino Acid	Anticodon	# tRNAs	tRNA Gene ID	W_ik_	Codon	‰ in CDS ^a^
Phe	GAA	3	LINF_070006450 * LINF_090016700 LINF_310035600	1	TTC	19.14
	AAA	0		0.54	TTT	10.32
Leu	CAG	2	LINF_090013100 LINF_230012100	1	CTG	38.12
	AAG	3	LINF_110009900 LINF_110010300 LINF_360074600	0.30	CTT	11.26
	UAA	1	LINF_240022200	0.04	TTA	1.64
	CAA	1	LINF_290025800	0.29	TTG	10.87
	UAG	1	LINF_340017300	0.12	CTA	4.68
	GAG	0		0.65	CTC	24.81
Ile	UAU	1	LINF_230011600	0.14	ATA	2.72
	AAU	3	LINF_240022300 LINF_240022400 LINF_340017100	0.44	ATT	8.37
	GAU	0		1	ATC	18.91
iMet	CAU	2	LINF_090013000 LINF_360074700	1	ATG	22.65
Met	CAU	2	LINF_110015000 LINF_340017200	1	ATG	
Val	CAC	2	LINF_090016400 LINF_090017100	1	GTG	37.53
	AAC	2	LINF_210018800 LINF_340017000	0.23	GTT	8.73
	UAC	1	LINF_230011700	0.15	GTA	5.45
	GAC	1	LINF_020010500	0.51	GTC	19.16
Ser	GCU	2	LINF_170013500 LINF_210010800	1	AGC	25.48
	CGA	1	LINF_290025900	0.83	TCG	21.18
	AGA	1	LINF_310035700	0.39	TCT	9.97
	UGA	1	LINF_340017400	0.29	TCA	7.37
	ACU	0		0.28	AGT	7.23
	GGA	0		0.64	TCC	16.32
Pro	AGG	2	LINF_210018700 LINF_360033400	0.34	CCT	8.83
	CGG	2	LINF_240010900 LINF_340042500	1	CCG	26.13
	UGG	1	LINF_360074800	0.40	CCA	10.48
	GGG	0		0.47	CCC	12.31
Thr	UGU	1	LINF_230012200	0.40	ACA	10.02
	CGU	2	LINF_300026300 LINF_340042400	1	ACG	25.01
	AGU	3	LINF_360019400 LINF_360019500 LINF_360033200	0.28	ACT	6.97
	GGU	0		0.70	ACC	17.47
Ala	CGC	2	LINF_110010000 LINF_110010200	1	GCG	45.36
	AGC	2	LINF_170013600 LINF_310011300	0.41	GCT	18.51
	UGC	1	LINF_330008700	0.45	GCA	20.40
	GGC	1	LINF_290019900	0.81	GCC	36.95
Tyr	GUA	3	LINF_340042300 LINF_360019300 LINF_360019600	1	TAC	20.00
	AUA	0		0.20	TAT	3.97
His	GUG	2	LINF_090016400 LINF_090017400 LINF_130017500 *	1	CAC	20.15
	AUG	0		0.33	CAT	6.59
Gln	CUG	3	LINF_160017000 LINF_240022100 LINF_360051500	1	CAG	33.20
	UUG	1	LINF_230011800	0.23	CAA	7.64
Asn	GUU	4	LINF_100020000 LINF_280009850 LINF_340042200 LINF_340042600	1	AAC	20.81
	AUU	1	LINF_230016900	0.26	AAT	5.45
Lys	CUU	4	LINF_030011800 LINF_100019900 LINF_210018900 LINF_330021200	1	AAG	28.08
	UUU	1	LINF_230012400	0.20	AAA	5.64
Asp	GUC	3	LINF_130017500 LINF_170013400 LINF_240024600	1	GAC	34.26
	AUC	0		0.43	GAT	14.57
Glu	UUC	1	LINF_090017100	0.24	GAA	11.63
	CUC	2	LINF_150015100 LINF_310019000	1	GAG	48.53
Cys	GCA	1	LINF_360074500	1	TGC	14.63
	ACA	0		0.27	TGT	3.93
Trp	CCA	1	LINF_230012600	1	TGG	10.79
Arg	ACG	4	LINF_050014900 LINF_070014000 LINF_110015000 LINF_230012000	0.32	CGT	10.31
	CCG	1	LINF_090017400	0.43	CGG	13.87
	UCG	1	LINF_230012300	0.23	CGA	7.54
	UCU	2	LINF_180013900 LINF_330008600	0.09	AGA	2.79
	CCU	1	LINF_330008500	0.18	AGG	5.70
	GCG	0		1	CGC	32.50
Gly	GCC	4	LINF_100020000 LINF_310018900 LINF_360033100 LINF_360033300	1	GGC	34.75
	CCC	1	LINF_110010100	0.34	GGG	11.87
	UCC	1	LINF_230012500	0.19	GGA	6.68
	ACC	1	LINF_210019700	0.35	GGT	12.11
SeC	UCA	1	LINF_060007300	NA	TGA	NA

# Number of tRNAs. ^a^ Determined from the coding sequences (CDS) of the 8532 *L. infantum* protein-coding genes annotated to date. * tRNA with atypical structure (see Figure 1).

## Data Availability

The proteome raw data generated in this study are publicly available at Zenodo repository (https://zenodo.org/records/18639527, accessed on 11 April 2026).

## Supplementary Materials

The following supporting information can be downloaded at https://www.mdpi.com/article/10.3390/genes17060620/s1, Supplementary File S1 is an Excel file containing tRNAs sequences, codon usage for every *L. infantum* protein-coding gene, tAI scores, relative protein abundances, and codon usage tables for the species analyzed in this study; Supplementary Figure S1 shows the structure of the intron-containing tRNA genes identified in this study.
