# Supplementary material for: Transfer RNA (tRNA) Genes, Codon Usage and Translational Efficiency in Leishmania infantum
Source: Genes (Basel). 2026 May 29;17(6):620. doi: 10.3390/genes17060620 (PMC13298570; doi:10.3390/genes17060620)
Supplement: Supplementary file 1 [file genes-17-00620-s001.zip › Supplementary Figure S1.pdf]

A

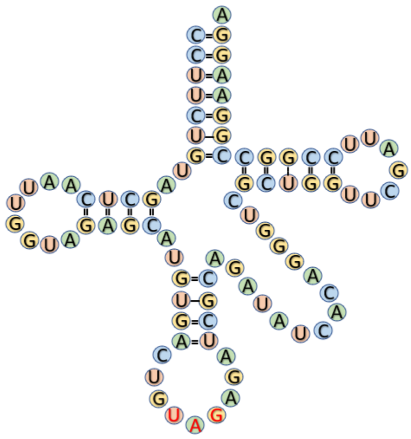

LINF_340042300 + intron

Sequence:
CCUUCUGUAGCUCAAUUGGUAGAGCAUGUGACUGUAGAgaucgcagauaUCACAGGGUCGCUGG
UUCGAUUCCGGCCGGAAGGA

B

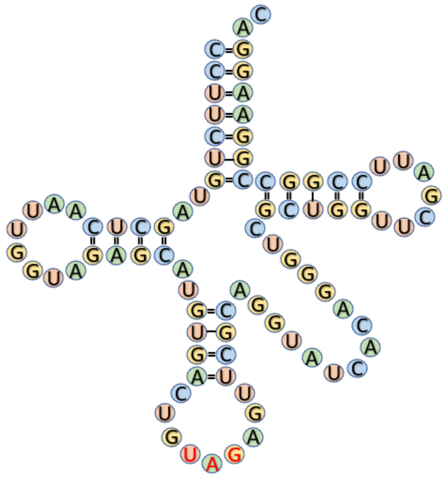

LINF_360019300+intron / LINF_360019600+intron

Sequence:
CCUUCUGUAGCUCAAUUGGUAGAGCAUGUGACUGUAGAguucgcagguaUCACAGGGUCGCUGG
UUCGAUUCCGGCCGGAAGGAC

Supplementary Figure S1. Predicted secondary structure for tRNAs including the intronic sequence (lowercase). (**A**) tRNA LINF_340042300. (**B**) tRNAs LINF_360019300 and LINF_360019600 (both have identical sequence).
